# An Improved Microaneurysm Detection Model Based on SwinIR and YOLOv8

**DOI:** 10.3390/bioengineering10121405

**Published:** 2023-12-08

**Authors:** Bowei Zhang, Jing Li, Yun Bai, Qing Jiang, Biao Yan, Zhenhua Wang

**Affiliations:** 1 College of Information Science, Shanghai Ocean University, Shanghai 201306, China; m220951661@st.shou.edu.cn (B.Z.); m210911495@st.shou.edu.cn (Y.B.); 2 Department of Ophthalmology, Eye Institute, Eye and ENT Hospital, Fudan University, Shanghai 201114, Chinabiao.yan@fdeent.org (B.Y.); 3 The Affiliated Eye Hospital, Nanjing Medical University, Nanjing 211166, China

**Keywords:** diabetic retinopathy, microaneurysm, deep learning, YOLOv8, SwinIR

## Abstract

Diabetic retinopathy (DR) is a microvascular complication of diabetes. Microaneurysms (MAs) are often observed in the retinal vessels of diabetic patients and represent one of the earliest signs of DR. Accurate and efficient detection of MAs is crucial for the diagnosis of DR. In this study, an automatic model (MA-YOLO) is proposed for MA detection in fluorescein angiography (FFA) images. To obtain detailed features and improve the discriminability of MAs in FFA images, SwinIR was utilized to reconstruct super-resolution images. To solve the problems of missed detection of small features and feature information loss, an MA detection layer was added between the neck and the head sections of YOLOv8. To enhance the generalization ability of the MA-YOLO model, transfer learning was conducted between high-resolution images and low-resolution images. To avoid excessive penalization due to geometric factors and address sample distribution imbalance, the loss function was optimized by taking the Wise-IoU loss as a bounding box regression loss. The performance of the MA-YOLO model in MA detection was compared with that of other state-of-the-art models, including SSD, RetinaNet, YOLOv5, YOLOX, and YOLOv7. The results showed that the MA-YOLO model had the best performance in MA detection, as shown by its optimal metrics, including recall, precision, F1 score, and AP, which were 88.23%, 97.98%, 92.85%, and 94.62%, respectively. Collectively, the proposed MA-YOLO model is suitable for the automatic detection of MAs in FFA images, which can assist ophthalmologists in the diagnosis of the progression of DR.

## 1. Introduction

Diabetic retinopathy (DR) is one of microvascular complications affecting the retina, caused by diabetes. It is also known as a major cause of blindness worldwide [1,2]. The pathogenesis of DR is tightly associated with an altered vessel structure due to increased blood glucose levels. Initially, DR presents as tiny dilations of capillaries, known as microaneurysms (MAs) [3,4]. MAs are primarily distributed in the inner nuclear layer and deep capillary plexus and are often an early clinical manifestation of various retinal and systemic diseases, including DR, retinal vein occlusion, and infections. In fundus images, MAs appear as small dots and a visible pathology at the early stages of DR. Therefore, the accurate detection of MA is crucial for the prevention, diagnosis, and treatment of DR [5].

**Figure 1:**
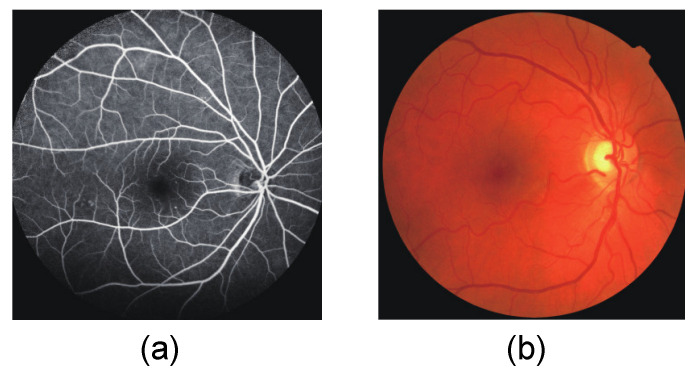
Fundus images. (**a**) FFA image; (**b**) NMFCS image.

The advancement of modern retinal imaging techniques, such as fundus fluorescein angiography (FFA) and non-mydriatic fundus photography (NMFCS), has improved the identification of MAs. FFA is a technique that uses the injection of a contrast agent to observe the retinal vessels. NMFCS refers to a non-invasive imaging technique of capturing retinal images through fundus photography. Figure 1 shows images obtained by FFA and NMFCS. The fundus images obtained by FFA exhibit higher contrast and clearer presentation of the features of the retinal structure compared to the images obtained by NMFCS. In the clinical practice, FFA is widely recognized as an important standard for visualizing the retinal vasculature and describing subtle vascular changes.

**Figure 2:**
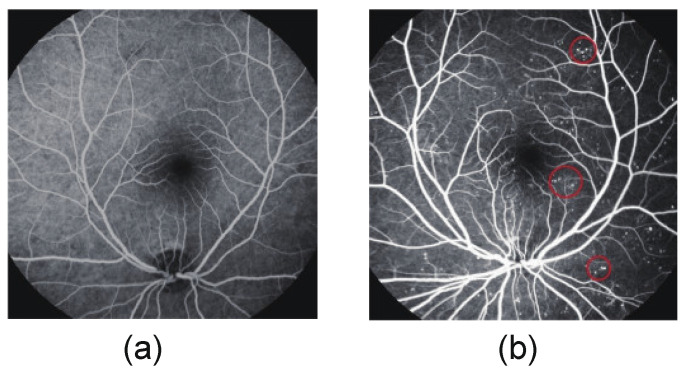
FFA images. (**a**) Normal FFA image; (**b**) FFA image with MAs.

Figure 2 shows a normal FFA image and an FFA image with microaneurysms. In the FFA image, MAs typically appear as round and bright spot-like structures with diameters ranging from 10 μm to 100 μm. MAs hold an important value in disease diagnosis and screening. Their objective quantitative evaluation is still limited as it requires manual detection by experienced technicians.

Over the last two decades, automatic detection models for MAs have rapidly developed based on deep learning. The convolutional neural network (CNN) is a deep learning algorithm that extracts features from images through multiple layers of convolution and pooling operations and utilizes the fully connected layers for classification or regression tasks. The CNN has achieved great success in the field of image processing and is widely used for object recognition and semantic segmentation. Object recognition using CNNs has great advantages such as high accuracy, application flexibility, automation, and real-time performance, providing support for practical applications such as SSD [6], RetinaNet [7], YOLOv5, YOLOv7 [8], and YOLOX [9]. Meanwhile, previous studies reported several segmentation models for the automatic detection of MAs. Liao et al. proposed a deep convolutional encoder–decoder network with a weighted dice loss for MA localization [10]. Xia et al. introduced a multi-scale model for detecting and classifying MAs using residual and efficient networks [11]. Chudzik et al. proposed a three-stage detection method as an alternative to the traditional five-stage MA detection. This study demonstrated successful transfer learning between small MA datasets [12]. Zhou et al. proposed a collaborative learning model based on a fine-tuning detection module in a semi-supervised manner to improve the performance of MA detection [13]. Xie et al. proposed a segmentation–emendation–resegmentation–verification framework to predict and correct detection errors in models, enhancing the detection of MAs [14]. Wang et al. utilized a region-based fully convolutional network (R-FCN) incorporating a feature pyramid network and an improved region proposal network for MA detection [15]. Guo et al. proposed a novel end-to-end unified framework for MA detection that utilizes multi-scale feature fusion and multi-channel bin loss [16]. Mateen et al. proposed a hybrid feature embedding approach using pre-trained VGG-19 and Inception-v3 for MA detection [17]. Kumar et al. trained a model of radial basis function neural network for MA detection [18]. Table 1 shows the strengths and weaknesses of the reported models for MA detection. The above-mentioned MA detection models based on deep learning have enhanced the efficiency of MA detection in FFA images. However, the tiny size of MAs, their low contrast with the background, and the lack of an annotated MA database still pose a great challenge for MA detection. Thus, further study is still required to design a novel detection method to enhance MA detection efficiency.

MAs are relatively small in size and often appear as tiny and blurry lesions in retinal images, which are particularly pronounced in low-resolution images. They are often similar to the pixels of blood vessels. Super-resolution reconstruction is an image processing technique that can enhance the spatial resolution and detail clarity of an image by recovering high-resolution details from a low-resolution image. The Swin Transformer [19] has shown great promise as it integrates the advantages of both CNN and Transformer. The Swin Transformer processes large images using a self-attention mechanism and models long-range dependencies with a shifted window scheme. An image restoration model, SwinIR [20], was designed based on the Swin Transformer. SwinIR could not only enhance the detail features of MAs, but also improve the visibility and discriminability of MAs in FFA images. Except for tiny MAs in FFA images, sample imbalance and loss of information are two problems to be solved that affect the accuracy and efficiency of MA detection. YOLOv8 is an object recognition algorithm that is characterized by its ability to perform object localization and classification in a single forward pass. YOLOv8 contains a backbone, a neck, and a head. The neck section utilizes the path aggregation network (PAN)–feature pyramid network (FPN) structure for feature fusion [21,22]. FPN constructs a multi-scale feature pyramid by adding lateral connections and up-sampling layers to capture rich semantic information and better detect objects of different sizes. PAN addresses the issue of feature propagation in FPN by aggregating and propagating features through horizontal and vertical paths. PAN–FPN combines the strengths of FPN and PAN to provide powerful feature representation capabilities. The backbone and neck sections of YOLOv8 draw inspiration from the design principles of YOLOv7 ELAN. The C3 structure of YOLOv5 is replaced with the C2f structure in YOLOv8, which has a richer gradient flow, allowing for a better capturing of image details and contextual information. Given its powerful identification efficiency, multi-scale feature fusion, and contextual information capturing, the proposed MA detection model was designed based on YOLOv8.

Therefore, an improved MA detection model for FFA images based on SwinIR and YOLOv8 is proposed, called MA-YOLO. The major contributions of this study proposing the new MA-YOLO model are as follows:

- SwinIR was used to reconstruct high-resolution FFA images, which could enhance the visibility and discriminability of MAs in FFA images.
- A detection layer was added to the YOLOv8 model, which could avoid feature information loss in shallow layers and improve the performance of MA detection.
- Transfer learning was utilized between high- and low-resolution images to expand the data samples and improve the generalization ability.
- Taking Wise-IoU as the bounding box regression loss, the loss function of MA-YOLO was improved, which could relieve the sample distribution imbalance problem and enhance the generalization performance.

In addition, the proposed MA-YOLO model could calculate the MA area in FFA images, which would assist ophthalmologists in assessing the progression of DR.

## 2. Materials and Methods

### 2.1. Materials

#### 2.1.1. Datasets

The experimental dataset used in this study contained two datasets. The first dataset was collaboratively constructed by the Nanjing Medical University-Affiliated Eye Hospital and includes 1200 FFA images (768 × 868 pixels) from 1200 eyes of DR patients (age range, 31–81 years). Image acquisition was performed using the Heidelberg retina angiograph (Heidelberg Engineering, Germany) device. To ensure data quality, the dataset excluded images that were blurry or overexposed because of environmental factors or equipment materials. 

The second dataset originated from a study conducted at the Persian Eye Clinic (Feiz Hospital) at the Isfahan University of Medical Sciences. The dataset includes 70 retinal images (576 × 720 pixels) from a total of 70 patients, with 30 images classified as normal, and 40 images representing different stages of abnormality. Prior to image collection, each patient underwent a comprehensive ophthalmic evaluation, which involved medical history assessment, applanation tonometry, slit-lamp examination, dilated fundus biomicroscopy, and ophthalmoscopy [23].

Based on the above datasets, a total of 1240 FFA images were selected as the experimental dataset. All images were resized to 768 × 768 pixels and annotated by clinical doctors with more than 10 years of clinical experiences. The 1240 FFA images were independently divided into 992 training FFA images, 124 validation FFA images, and 124 test FFA images.

#### 2.1.2. Implementation

The hardware configuration used for the experiment was Ubuntu 20.04.5, 2GPUs, GPU NVIDIA RTX 2080ti and 1 GPU memory (11 GB). As software, we used the deep-learning framework Pytorch 2.0.0 and the programming language python 3.8.

#### 2.1.3. Evaluation Metrics

Five metrics were calculated to estimate the performance of MA detection [24], i.e., recall (Re), precision (Pre), F1 score (F1), average precision (AP), and frames per second (FPS).

(1) Re=TP/(TP+FN)

(2) Pre=TP/(TP+FP)

(3) F1=2×Pre×Re/(Pre+Re)

(4) $$AP=\int_{0}^{1}\;Pre(Re)dRe$$

(5) $$\mathrm{FPS}\;=\frac{\;\mathrm{frameNum}\;}{\;\mathrm{elapsedTime}\;}$$

TP, FP, and FN denote true positive regions, false positive regions, and false negative regions, respectively. frameNum is the number of FFA images inputted into the detection model; elapsedTime is the time consumed by the detection model. Re and Pre are the proportion of correct predictions in all MAs and the proportion of real MAs in the samples predicted as MAs. F1 is a balanced metric determined by precision and recall. AP is the area under the precision–recall (PR) curve, obtained by plotting recall on the *x*-axis and precision on the *y*-axis, based on a set of precision and recall values calculated at different thresholds. FPS is the number of FFA images inferred per second.

### 2.2. Methods

**Figure 3:**
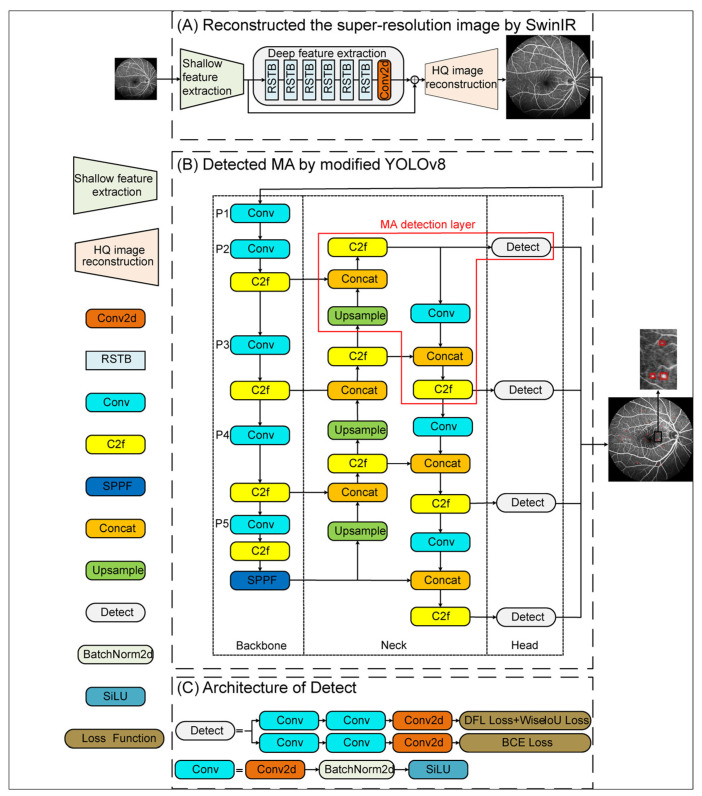
Flowchart of MA-YOLO.

Figure 3 illustrates the flowchart of the proposed MA-YOLO model. In this model, SwinIR was used for the super-resolution reconstruction of FFA images (Figure 3A). The MA detection layer was added to modify YOLOv8 (Figure 3B), and transfer learning was applied to increase the amount of data. The Wise-IoU loss was utilized to enhance the detection capability (Figure 3C).

#### 2.2.1. Super-Resolution FFA Image Reconstruction Based on SwinIR

MAs are small in size and usually appear as tiny and blurry structures in FFA images. The subtle features of MAs are easily lost in low-resolution images. Therefore, reconstructing high-resolution images is helpful for MA detection in FFA images. Here, SwinIR was employed to perform the super-resolution reconstruction of FFA images.

SwinIR, an image restoration technique, contains three modules, i.e., shallow feature extraction, deep feature extraction, and HQ image reconstruction (high-quality image reconstruction modules). The shallow feature extraction module uses a convolution layer to extract shallow features, which are directly transmitted to the reconstruction module and preserve low-frequency information. The deep feature extraction module is mainly composed of residual Swin Transformer blocks (RSTB), each of which utilizes several Swin Transformer layers for self-attention and cross-window interaction. Additionally, a convolution layer was incorporated at the end of the block to enhance features. A residual connection was utilized to establish a shortcut for feature aggregation. Finally, both shallow and deep features were transmitted to the HQ image reconstruction module, which used the sub-pixel convolution layer [25] to up-sample the features for high-quality image reconstruction.

**Figure 4:**
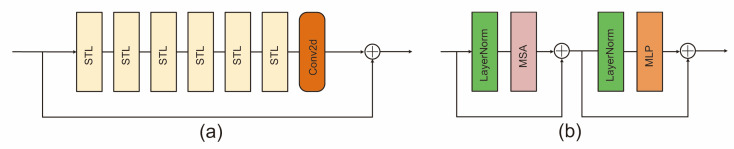
Architecture of RSTB and STL. (**a**) Residual Swin Transformer block (RSTB); (**b**) Swin Transformer layer (STL).

Figure 4 illustrates the structure of the residual Swin Transformer block and the Swin Transformer layer.

Based on SwinIR, the original FFA images with a size of 768 × 768 pixels were reconstructed into super-resolution FFA images with a size of 1536 × 1536 pixels and 2304 × 2304 pixels, respectively, which effectively enhanced the detail features of MAs.

#### 2.2.2. YOLOv8 Modified by MA Detection Layer and Transfer Learning

During the down-sampling of the convolution layers of YOLOv8, the regions containing MAs become blurred, and it was difficult to accurately localize the MAs. Thus, down-sampling convolution caused the loss of small features and missed and false MA detection.

**Figure 5:**
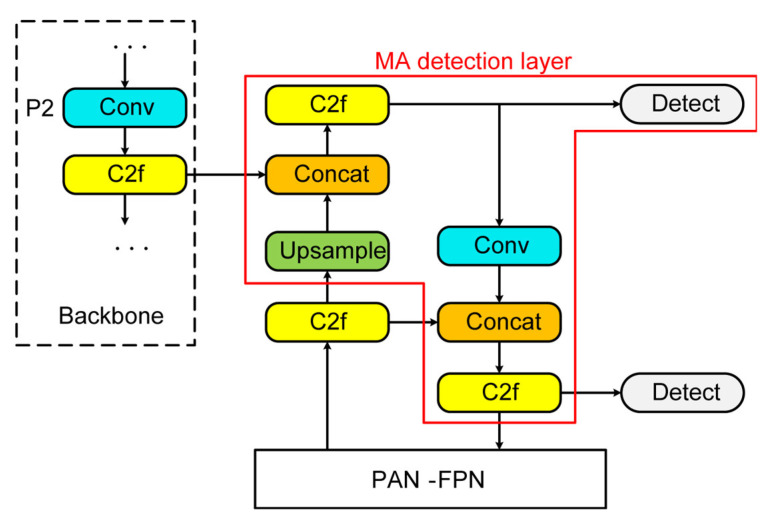
Architecture of the MA detection layer.

Here, an MA detection layer was introduced into the neck and head of YOLOv8 to handle shallow feature maps from the P2 layer of the backbone network and integrate them into the PAN–FPN structure. The architecture of the MA detection layer is shown in Figure 5. The MA detection layer up-sampled deep-level feature maps with stronger semantic features from the FPN structure and then concatenated them with shallow-level feature maps outputted by the P2 layer of the backbone network, enhancing the semantic expression of the shallow-level features. After feature extraction by the c2f module, the resulting features were passed into the added detection head. Simultaneously, the MA detection layer down-sampled the obtained feature maps using convolution, concatenated them with the deep-level feature maps, and then underwent another feature extraction by the c2f module. This process integrated the feature information extracted from the shallow levels into the PAN structure, enhancing the model’s localization capability at various scales. Based on the modified YOLOv8, small MA features could be obtained, and the accuracy of MA detection could be enhanced.

Due to the limited amount of MA data, few annotated samples are available for training and evaluation, and it is a challenge to construct accurate and reliable models. Here, transfer learning was used to modify YOLOv8. Transfer learning [26] is a machine learning technique that leverages the knowledge gained from one task to improve its performance on different but related tasks. Transfer learning is usually used to transfer pre-trained models or features.

**Figure 6:**
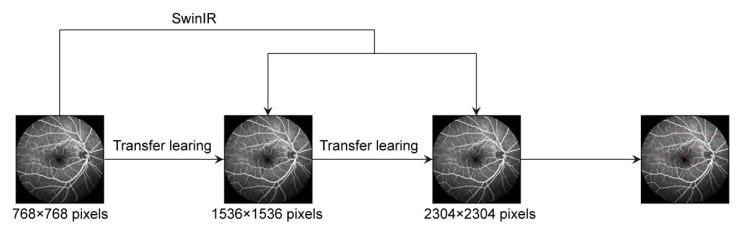
Flowchart of transfer learning.

Using transfer learning, three different datasets were leveraged while training the model, including the original MA images with a size of 768 × 768 pixels and two super-resolution reconstructed images with a size of 1536 × 1536 and 2304 × 2304 pixels. Figure 6 shows the flowchart of transfer learning applied to these three different datasets. Based on the original MA images of 768 × 768 pixels, the detection model was pre-trained, and the learned knowledge was retained. Based on the super-resolution reconstructed images of 1536 × 1536 pixels, the detection model was transferred and fine-tuned. The learned knowledge was updated. Based on the super-resolution reconstructed images of 2304 × 2304 pixels, the detection model was transferred and fine-tuned, and the learned knowledge was updated again.

#### 2.2.3. Loss Function Optimization Based on Wise-IoU

The loss function of the official YOLOv8 consists of two components: classification and regression. For classification, binary cross-entropy loss (*BCEL*) is used as the loss function, while for regression, distribution focal loss (*DFL*) [27] and *CIoU* [28] bounding box regression loss (*CIoUL*) are incorporated.

The loss function of YOLOv8 is represented as

(6) $$f_{\mathrm{loss}\;}=\lambda_{1}f_{BCEL}+\lambda_{2}f_{DFL}+\lambda_{3}f_{CIoUL},$$

On the basis of the official YOLOv8 weight parameter settings, the weight parameters $\lambda_{1}$, $\lambda_{2}$, and $\lambda_{3}$ were always set to 0.05, 0.15, and 0.75, respectively.

*BCEL* is defined as

(7) $$f_{BCEL}=\;\mathrm{weight}\;[\;\mathrm{class}\;](-x[\;\mathrm{class}\;]+log(\sum_{j}\;exp(x[j]))),$$

where class is the number of categories, weight [ class ] denotes the weight for each class, and x is the probability value after sigmoid activation.

*DFL* is an optimization of the focal loss function, which generalizes the discrete results of classification into continuous results through integration, denoted as

(8) $$f_{DFL}\left(S_{i},S_{i+1}\right)=-\left(\left(y_{i+1}-y\right)\log\left(S_{i}\right)+\left(y-y_{i}\right)\log\left(S_{i+1}\right)\right),$$

where $y_{i},y_{i+1}$ represent the values from the left and right sides near the consecutive labels y, satisfying $y_{i}<y<y_{i+1},y=\sum_{i=0}^{n}\;P\left(y_{i}\right)y_{i\;},\;P\left(y_{i}\right)=S_{i}$; P can be implemented through a softmax layer.

According to the calculation of the overlap between the ground truth box and the predicted box and the differences in center point distance and aspect ratio, *CIoUL* reflects the similarity and accuracy of two bounding boxes and is defined as

(9) $$f_{CIoUL}=1-IOU+\frac{\rho^{2}\left(b,b^{gt}\right)}{c^{2}}+\alpha v,$$

(10) $$v=\frac{4}{\pi^{2}}{\left(\arctan\frac{w^{gt}}{h^{gt}}-\arctan\frac{w}{h}\right)}^{2},$$

(11) $$\alpha =\frac{v}{1-IOU+v},$$

where $\frac{\rho^{2}\left(b,b^{gt}\right)}{c^{2}}$ is the distance between the centers of the target box and the prediction box, c is the distance between the diagonal points of the smallest enclosing box, $w^{gt}$ and $h^{gt}$ represent the size of the target box, and w and h represent the size of the prediction box. However, *CIoUL* ignores the issue of sample distribution imbalance and presents limitations in relation to small MAs and in the presence of a large background noise.

Here, *CIoUL* was replaced with Wise-IoU [29] bounding box regression loss. The Wise-IoU loss function uses a dynamic focusing mechanism to evaluate the quality of the anchor box, where an “outlier” is used to avoid excessive penalties for geometric factors (such as distance and aspect ratio). Additionally, the Wise-IoU loss borrows the idea of focal loss, using a focus coefficient constructed to reduce the contribution of samples easy to evaluate to the loss value. The Wise-IoU loss function is defined as

(12) $$L_{WIoU}=\beta^{\gamma }\exp\left(\frac{{\left(x-x_{gt}\right)}^{2}+{\left(y-y_{gt}\right)}^{2}}{{\left(W_{g}^{2}+H_{g}^{2}\right)}^{*}}\right)L_{IoU},$$

(13) $$\begin{array}{c} \beta =\frac{L_{IoU}^{*}}{\bar{L_{IoU}}} \end{array}\;\;\in [0,+\infty ),$$

where $w_{g}$, Hg are the size of the smallest enclosing box, x and y represent the coordinate values of the prediction box, $x_{gt}$ and $y_{gt}$ represent the coordinate values of the ground truth, γ is an adjustable hyperparameter, set to 0.5, and β indicates the degree of abnormality of the prediction box (a small degree of abnormality means that the quality of the anchor box is high). Therefore, β can assign small gradient gains to prediction boxes with large outliers, effectively reducing the harmful gradients of low-quality training samples.

## 3. Results

To evaluate the detection performance of the MA-YOLO model, two comparative experiments were performed. Experiment one was an ablation experiment, where the MA-YOLO model was compared with YOLOv8 with different settings. Experiment two was a comparative experiment, where the detection performance of the MA-YOLO model was compared with that of other models, including SSD, RetinaNet, YOLOv5, YOLOX, and YOLOv7.

### 3.1. Ablation Experiment

**Figure 7:**
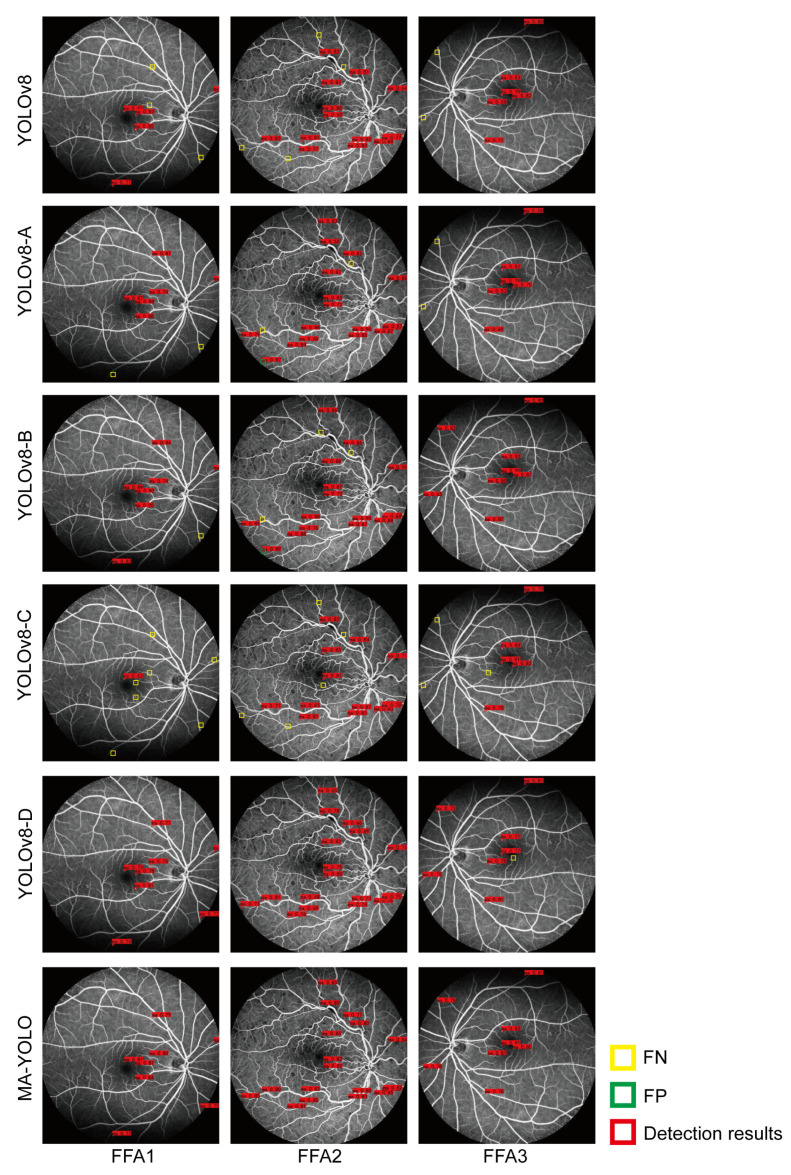
MA detection by the YOLOv8 model with different settings, where the red boxes represent the detection results with a confidence score greater than 0.5, the yellow boxes represent missed detection, and the green boxes represent false positive detection.

The proposed MA-YOLO model was compared to YOLOv8 with different settings, including YOLOv8 with SwinIR (YOLOv8-A), YOLOv8 with SwinIR and transfer learning (YOLOv8-B), YOLOv8 with the Wise-IoU loss function (YOLOv8-C), and YOLOv8 with the MA detection layer (YOLOv8-D). Figure 7 and Table 2 show the results of MA detection and the evaluation metrics for MA detection by YOLOv8 with different settings.

Based on Table 2, MA-YOLO provided the best scores of Re, Pre, F1, and AP in MA detection, which were 88.23, 97.98, 92.85, and 94.62, respectively. However, due to the addition of the MA detection layer, FPS was 1.51, which was lower than that of YOLOv8. Compared with the YOLOv8 model, YOLOv8-A allowed improving the scores of Re, F1, and AP, which were 83.44 (2.63↑), 84.46 (1.1↑), and 83.46 (1.37↑), respectively. YOLOv8-B also allowed improving the scores of Re, Pre, F1, and AP, which were 85.22 (4.41↑), 88.07 (2↑), 86.62 (3.26↑), and 84.13 (2.04↑), respectively. The same was observed for YOLOv8-C with scores of Re, Pre, F1, and AP of 84.65 (3.84↑), 86.73 (0.66↑), 85.68 (2.32↑), and 87.29 (5.2↑), respectively. Also YOLOv8-D allowed improving the scores of Re, Pre, F1, and AP, which were 86.15 (5.34↑), 93.19 (7.12↑), 89.53 (6.17↑), and 88.67 (6.58↑), respectively.

As shown in Figure 7, MA-YOLO provided the best performance for MA detection, with few missed and false detection results. We observed some false MA detection with the YOLOv8-A and YOLOv8-B models and some missed MA detection with the YOLOv8, YOLOv8-A, YOLOv8-B, YOLOv8-C, and YOLOv8-D models.

**Figure 8:**
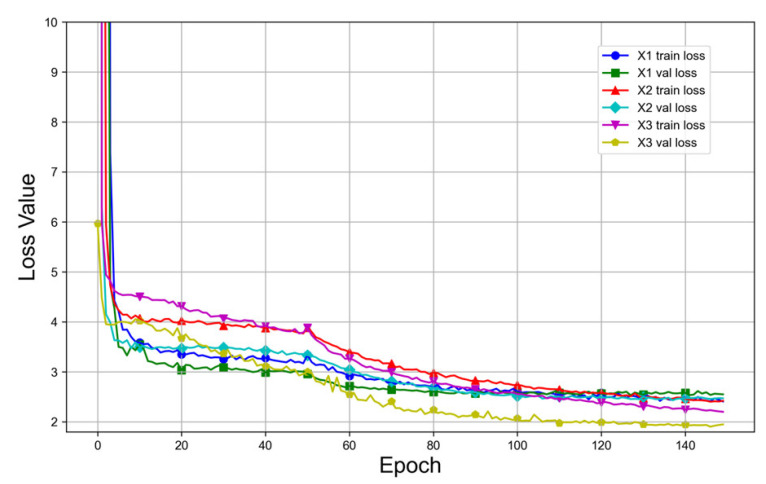
Comparison of the loss curves between the original images and the super-resolution images.

**Figure 9:**
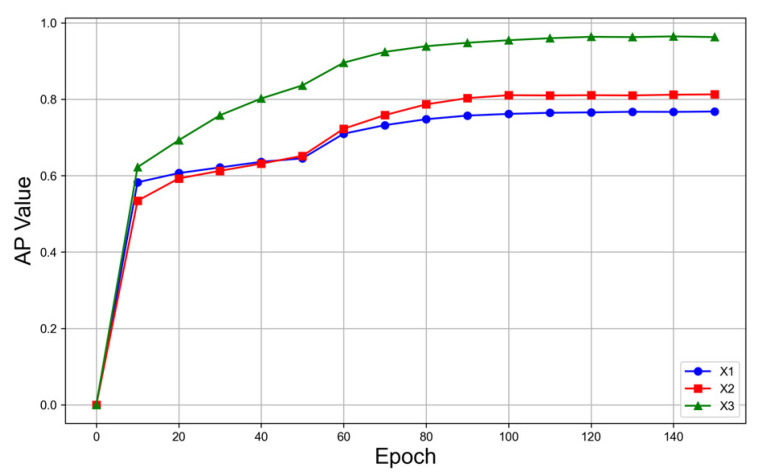
Comparison of the AP curves between the original images and the super-resolution images.

Figure 8 and Figure 9 illustrate the comparison of the loss curves and AP curves of the validation set between the original images and the super-resolution FFA images, where X1 denotes the original images with a size of 768 × 768 pixels, X2 the super-resolution images with a size of 1536 × 1536 pixels, and X3 the super-resolution images with a size of 2304 × 2304 pixels. Based on Figure 8 and Figure 9, it is evident that the model trained with super-resolution images demonstrated superior convergence trends and detection performance compared to the model trained with the original images.

### 3.2. Comparison Experiment

To evaluate the performance of MA detection, the proposed MA-YOLO model was compared with other models, including SSD, RetinaNet, YOLOv5, YOLOX, and YOLOv7. SSD is a classic one-stage object recognition algorithm, and its high detection speed makes it highly valuable for practical applications. RetinaNet has enhanced the ability of object recognition models to detect small objects by introducing focal loss. YOLOv5, YOLOX, and YOLOv7 are all part of the series of YOLO algorithms, representing newer models introduced in recent years. In addition, two reports were also selected to evaluate the proposed model’s performance in detecting MAs [24,30].

**Figure 10:**
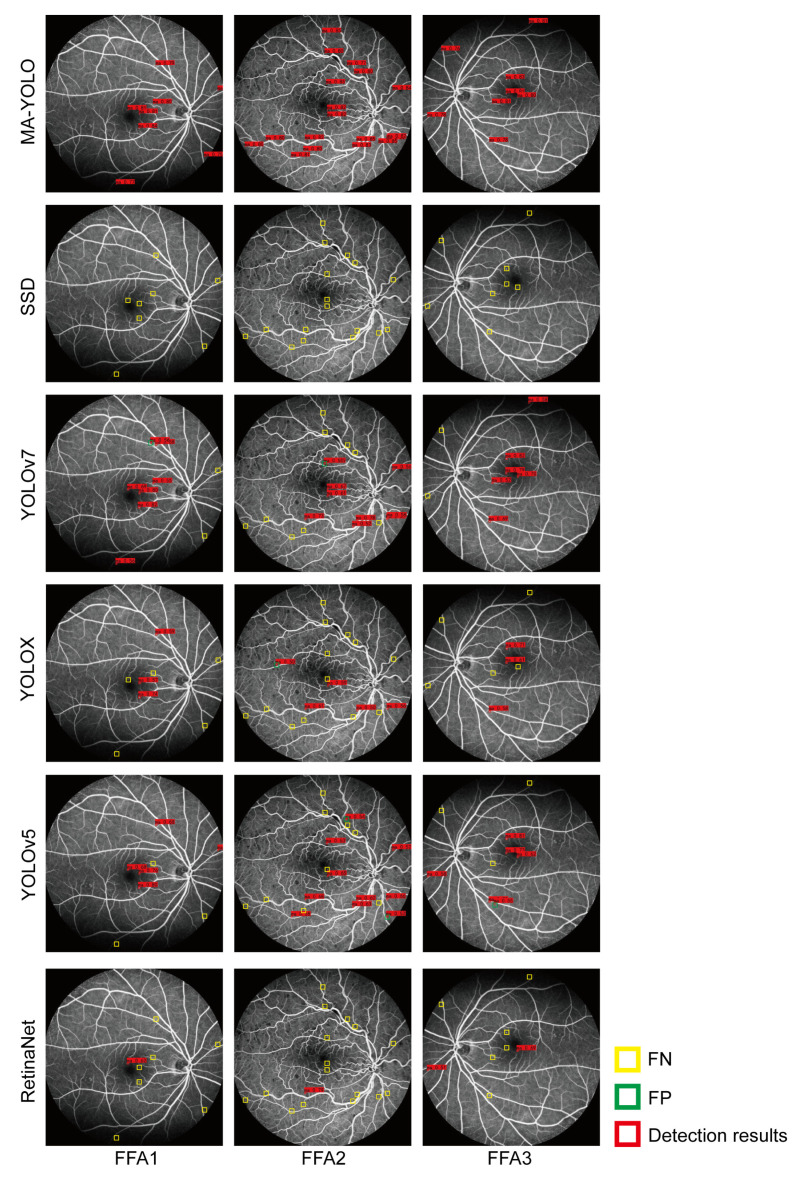
MA detection results by different models.

Table 3 and Table 4 show the comparison of MA detection performance and tuning parameters during the training phase among different models. Figure 10 shows the MA detection results of different models, where the red boxes represent the detection results with a confidence score greater than 0.5, the yellow boxes indicate missed detection, and the green boxes represent false positive detection. Table 5 shows the comparison of the MA detection performance of different object recognition models reported in various studies.

According to Figure 10 and Table 3 and Table 4, the detection results of MA-YOLO were close to ground truth. Part of the background was mistakenly detected by the YOLOv5, YOLOX, and YOLOv7 models. We observed some missed MA detections by the SSD, RetinaNet, YOLOv5, YOLOX, and YOLOv7 models. MA-YOLO achieved the highest Re, Pre, F1, and AP scores compared to the other models and a higher FPS score than RetinaNet. According to Table 5, the detection performance of MA-YOLO was superior to that of the other examined methods.

### 3.3. Calculation of the MA Region

**Figure 11:**
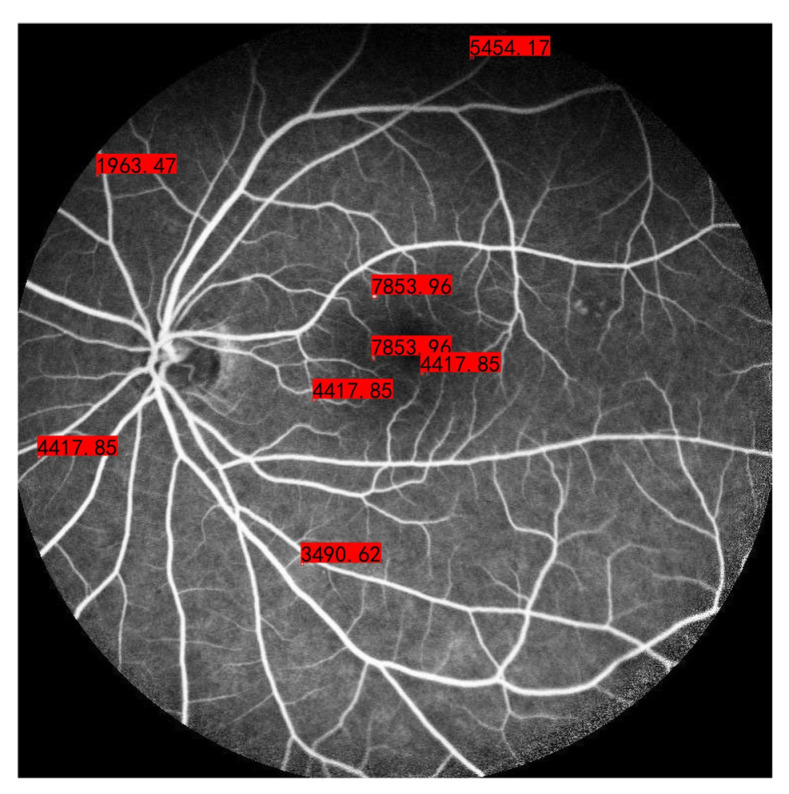
Calculation of the MA region.

In addition to MA detection, the MA area was calculated by the inscribed circle within the detection bounding box. The MA area could serve as an indicator to assess the progression of DR. Figure 11 shows the calculation results for the MA area, where the unit of measure is $\mu m^{2}$. The MA area was calculated in FFA images captured by a Heidelberg retina angiograph with a 55° lens and included 768 × 768 pixels, with each pixel corresponding to 25 μm in reality.

## 4. Discussion

Microaneurysms (MA) are recognized as the earliest symptom of DR that leads to retinal blood injury. The detection of MAs within FFA images facilitates the early DR diagnosis and prevents vision loss. However, MAs are extremely small, and their contrast with the surrounding background is very subtle, which makes MA detection challenging. MA objective and quantitative evaluation is still limited because it requires manual detection by experienced technicians. This study has great potential by allowing the detection and precise localization of MAs in retinal images. The proposed model’s outputs can be directly utilized by ophthalmologists for MA detection, eliminating the need for manual intervention. It contributes to the automation of MA detection, effectively guiding and assisting ophthalmologists in the treatment and elimination of MAs. The MA area can serve as an indicator to assess the progression of DR. A large area indicates a more severe condition, requiring more proactive treatment and management measures. Changes in the MA area can provide information about the stability or deterioration of the condition. The proposed model can be used to calculate the MA area in FFA images. By regularly calculating the MA area, the progression of DR and the effectiveness of treatments can be monitored.

Due to the addition of the MA detection layer and the model’s handling of higher-resolution images, the improved performance of MA detection may result in a decrease in the speed of MA detection to some extent. In addition, this proposed model was only applied on a limited dataset, and the validation of the model performance still requires its application on independent data from different patient cohorts across various medical centers. Future research will concentrate on addressing the aforementioned issues, by quantifying the model’s uncertainty [31,32], enhancing the detection speed through parameter pruning, and conducting an in-depth analysis of the model’s interpretability [33,34,35].

## 5. Conclusions

This study proposes the MA-YOLO model for the automatic detection of MAs in FFA images, based on image super-resolution reconstruction for data enhancement. This method can accurately and effectively detect MAs in FFA images. The algorithm utilized SwinIR for image super-resolution reconstruction, transforming the size of FFA images from 768 × 768 pixels to 1536 × 1536 pixels and 2304 × 2304 pixels. By reconstructing low-resolution FFA images, the details of MAs as well as their visibility and discriminability in the images were improved. Based on these improvements, the structure and loss function of the YOLOv8 model were further optimized. To address the challenges of extracting small features and the loss of feature information for MA detection, an MA detection layer was added to enhance feature extraction. Additionally, transfer learning was conducted between high-resolution and low-resolution datasets to enhance the model’s generalization. The Wise-IoU bounding box regression loss was employed to avoid excessive penalization due to geometric factors, improving the model’s generalization performance and addressing the problem of sample distribution imbalance. In addition, the MA-YOLO model can be used to calculate the MA area in FFA images to assist ophthalmologists in assessing the progression of DR.

Using the FFA dataset, ablation experiments were conducted to analyze and validate the effectiveness of the proposed model in the automatic detection of MAs. Furthermore, the proposed model was compared with five detection algorithms, i.e., SSD, YOLOv5, YOLOv7, YOLOX, and RetinaNet. The results showed that the proposed model outperformed these algorithms in terms of MA detection. The MA-YOLO model is thus a prospective approach for the early diagnosis of DR. In the future, the model will be further improved by incorporating more feature learning capabilities to achieve a higher detection speed.

## Figures and Tables

**Table 1:** Strengths and weaknesses of different models for MA detection.

Model	Strength	Weakness
U-net + DiceLoss + Activation function with long tail [10]	Increasing discrimination ability of probability maps	Missed detection of low-contrast MAs
U-net + residual learning + EfficientNet [11]	Improving segmentation performance by adding a classification network	Structural complexity and time-consuming calculation
U-net + BN layers + Dice coefficient function [12]	Simplifying MA extraction using a three-stage method	Presence of a large number of patches and inefficient detection
U-net + semi-supervised learning [13]	Reduction of the reliance on data labeling	Weak learning performance for MA features
SESV framework + DeepLabv3+ [14]	Presenting a high level of versatility, could be extended to other networks	High spatial and computational complexity and high cost of training time
R-FCN [15]	Improving the ability to detect objects at different scales	MA detection was compromised by the absence of annotated images
L-Seg [16]	Prevention of information loss by multi-scale feature fusion	Serious misclassification problem in MA detection
VGG-19 + Inception-v3 [17]	Obtaining data correlation in original feature space using feature embedding	Sample distribution imbalance was neglected
Radial basis function neural network [18]	Enhancement of MA extraction by removing morphological structures inside the retinas	Morphological structure removal made MA extraction cumbersome

**Table 2:** Comparison of the MA detection performance between YOLOv8 models with different settings.

Model	Improvement Strategy				Re (%)	Pre (%)	F1 (%)	AP (%)	FPS (It/s)
	SwinIR	Transfer Learning	Wise-IoU	MA Detection Layer					
YOLOv8					80.81 ± 0.03	86.07 ± 0.03	83.36 ± 0.03	82.09 ± 0.03	16.11 ± 0.02
YOLOv8-A	✓				83.44 ± 0.01	85.50 ± 0.04	84.46 ± 0.04	83.46 ± 0.09	1.99 ± 0.05
YOLOv8-B	✓	✓			85.22 ± 0.12	88.07 ± 0.14	86.62 ± 0.11	84.13 ± 0.10	1.99 ± 0.04
YOLOv8-C			✓		84.65 ± 0.07	86.73 ± 0.06	85.68 ± 0.08	87.29 ± 0.12	16.11 ± 0.01
YOLOv8-D				✓	86.15 ± 0.05	93.19 ± 0.09	89.53 ± 0.06	88.67 ± 0.03	12.79 ± 0.04
MA-YOLO	✓	✓	✓	✓	88.23 ± 0.11	97.98 ± 0.06	92.85 ± 0.09	94.62 ± 0.06	1.51 ± 0.03

**Table 3:** Comparison of the MA detection performance among different models.

Model	Re (%)	Pre (%)	F1 (%)	AP (%)
SSD	32.77 ± 0.05	76.30 ± 0.07	45.85 ± 0.16	51.53 ± 0.06
RetinaNet	71.32 ± 0.03	70.99 ± 0.14	71.15 ± 0.09	72.04 ± 0.05
YOLOv5	69.62 ± 0.05	71.53 ± 0.15	70.56 ± 0.02	71.57 ± 0.07
YOLOX	60.72 ± 0.12	67.04 ± 0.06	63.72 ± 0.13	63.33 ± 0.05
YOLOv7	68.18 ± 0.02	77.78 ± 0.07	72.66 ± 0.08	76.48 ± 0.02
MA-YOLO	88.23 ± 0.11	97.98 ± 0.06	92.85 ± 0.09	94.62 ± 0.06

**Table 4:** Tuning parameters and time of execution of different models.

Model	Epochs	Freeze Backbone Epochs	UnFreeze Epochs	Batch Size	Optimizer	Initial Learning Rate	Learning Rate Decay	FPS (it/s)
SSD	150	50	100	4	SGD	0.01	cosine annealing	22.93 ± 0.02
RetinaNet	150	50	100	4	SGD	0.01	cosine annealing	0.53 ± 0.02
YOLOv5	150	50	100	4	SGD	0.01	cosine annealing	16.85 ± 0.04
YOLOX	150	50	100	4	SGD	0.01	cosine annealing	13.74 ± 0.02
YOLOv7	150	50	100	4	SGD	0.01	cosine annealing	33.86 ± 0.04
MA-YOLO	150	50	100	4	SGD	0.01	cosine annealing	1.51 ± 0.03

**Table 5:** Comparison of MA detection performance among different studies.

Model	Re (%)	Pre (%)	F1 (%)	AP (%)
Rohan [30]	78.9	86.7	82.61	81.3
Gao [24]	89.77	87.13	88.51	88.92
MA-YOLO	88.23	97.98	92.85	94.62

## Data Availability

The data used to support the findings of this study are included within the article.
